# Point spread function estimation with computed wavefronts for deconvolution of hyperspectral imaging data

**DOI:** 10.1038/s41598-024-84790-6

**Published:** 2025-01-03

**Authors:** Miroslav Zabic, Michel Reifenrath, Charlie Wegner, Hans Bethge, Timm Landes, Sophia Rudorf, Dag Heinemann

**Affiliations:** 1 Hannover Centre for Optical Technologies (HOT), Leibniz University Hannover, Hannover, Germany; 2 Institute of Horticultural Production Systems, Leibniz University Hannover, Hannover, Germany; 3 HAIP Solutions GmbH, Hannover, Germany; 4 PhoenixD Cluster of Excellence, Leibniz University Hannover, Hannover, Germany; 5 Institute of Cell Biology and Biophysics, Leibniz University Hannover, Hannover, Germany

**Keywords:** Optics and photonics, Imaging and sensing

## Abstract

Hyperspectral imaging (HSI) systems acquire images with spectral information over a wide range of wavelengths but are often affected by chromatic and other optical aberrations that degrade image quality. Deconvolution algorithms can improve the spatial resolution of HSI systems, yet retrieving the point spread function (PSF) is a crucial and challenging step. To address this challenge, we have developed a method for PSF estimation in HSI systems based on computed wavefronts. The proposed technique optimizes an image quality metric by modifying the shape of a computed wavefront using Zernike polynomials and subsequently calculating the corresponding PSFs for input into a deconvolution algorithm. This enables noise-free PSF estimation for the deconvolution of HSI data, leading to significantly improved spatial resolution and spatial co-registration of spectral channels over the entire wavelength range.

## Introduction

Imaging spectroscopy, nowadays more commonly referred to as hyperspectral imaging (HSI)^1^, is a valuable tool for a wide range of applications, such as remote sensing, precision agriculture, food quality inspection, medical diagnosis, and material analysis, among others^2^. By capturing images across a wide range of wavelengths, HSI reveals additional spectral information beyond the capabilities of conventional RGB or grayscale imaging. However, due to the use of a large wavelength range, HSI systems often suffer from chromatic and other optical aberrations that can degrade image quality and reduce spatial resolution.

To address this problem, deconvolution algorithms have been utilized to improve spatial resolution in HSI systems^3–6^. In image processing, deconvolution is the numerical reversal of image blurring that occurs during image acquisition due to optical aberrations. Deconvolution presupposes the retrieval of the point spread function (PSF) of the imaging system, which is a crucial step in the process. A PSF represents the response of an optical system to a point source. The most straightforward way to measure the PSF of an HSI system is by imaging point-like samples such as illuminated metal spheres or pinholes with a size smaller than the diffraction limit of the system under investigation^7,8^. However, due to the required small size of point-like sources, the signal-to-noise ratio can be quite low, which can prohibit successful PSF measurement in spectral regions with low sensitivity of the HSI system. An alternative approach for PSF estimation is the simulation of the optical system^9–11^. This can only be done if all the components of the system are known, which is usually not the case for commercial devices from the user’s point of view. Another very promising approach for PSF estimation is analyzing the response of edge or random noise targets^12–14^. However, current methods can be quite extensive in terms of image processing, as a regularization step is usually required to ensure the similarity of neighboring PSFs.

A user-friendly PSF estimation method, when combined with image deconvolution, has the potential to significantly enhance the image quality of HSI systems without additional hardware investments. This approach is particularly advantageous for the development of systems with intrinsically suboptimal optical designs, such as low-cost and miniaturized configurations and could thus promote the widespread use of HSI systems.

**Fig. 1:**
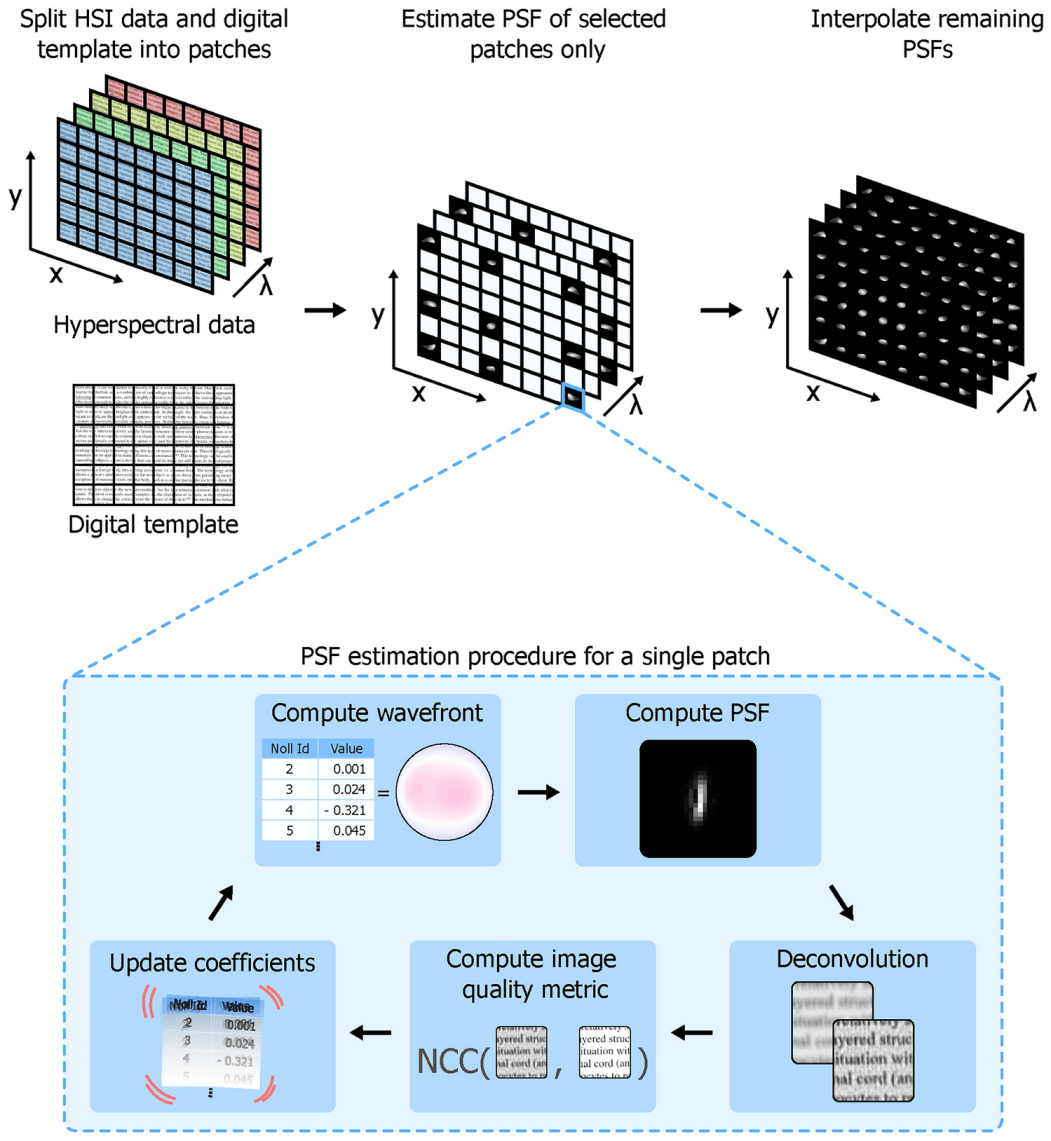
Basic concept for PSF estimation. HSI data and a digital ground truth template are divided into smaller patches to account for the spatial and spectral variation of the PSF. PSF estimation is performed for each patch individually. Due to the expected smooth transition of PSF shapes in the spatial and spectral directions, PSFs are estimated for a selected set of key patches and interpolated for the remaining patches. For the PSF estimation procedure for a single patch, an initial wavefront is derived from a list of Zernike polynomial coefficients. The corresponding PSF is then calculated and used to deconvolve the input patch. The deconvolved patch is compared with the digital template by computing the NCC. Based on the NCC value, the Zernike polynomial coefficients are adjusted by introducing a small random variation. If the NCC has improved, the adjustments are made to the current coefficient values; otherwise, the adjustments are applied to the previous coefficient values. Using these updated coefficients, a new wavefront is computed, and the process is repeated until the optimization algorithm’s stopping condition is met, resulting in an accurate PSF estimate for the current patch.

Here, we propose a novel method for PSF estimation in HSI systems based on computed wavefronts. Our method employs a simulated annealing optimization algorithm to computationally estimate the PSF. During each iteration of the optimization, the shape of a computed wavefront is modified, and the corresponding PSF is calculated. This PSF is then used for the deconvolution of the input image. The quality of the resulting deconvolved image is evaluated by computing the normalized cross-correlation (NCC) between the deconvolved image and a digital ground truth template. This process continues until the simulated annealing algorithm reaches its termination condition, thereby maximizing the NCC and generating an accurate PSF estimation of the imaging system and a deconvolved image with improved spatial resolution. Since the PSF is spectrally and spatially variant, this estimation process is applied to several spectrally and spatially separated patches of the entire dataset. The remaining PSFs are obtained by interpolating the computed wavefronts between all patches and calculating the corresponding PSFs from the interpolated wavefronts. Figure 1 illustrates the basic concept of our method. These obtained PSFs are then used to deconvolve entire HSI datasets from subsequent acquisitions. Additionally, the estimated PSFs are validated against experimentally acquired PSFs, obtained using a fiber-based array of point-like light sources.

## Related work

Deconvolution techniques have been extensively studied for various imaging systems and can be categorized into non-blind, blind, and semi-blind approaches. A non-blind approach is applicable when the PSF is fully known. Blind techniques are employed in the absence of a priori knowledge of the PSF. Semi-blind approaches are used when partial information about the PSF is available, which reduces the range of possible PSFs. The work we present here can be considered a semi-blind method. We use the assumption that blurring in the HSI images occurs only due to typical optical aberrations that can be described with Zernike polynomials. Based on this assumption, we model the PSF using a computed wavefront expressed as a set of Zernike polynomials, thereby constraining the solution space of possible PSFs. Additionally, we utilize a ground truth image that, while not providing the PSF directly, still offers a priori knowledge that aids in the PSF estimation process. There are already several deconvolution techniques for spatially varying PSFs published, as reviewed in^15–19^.

Here, we highlight the most closely related works. Brauers et al. proposed a method for direct PSF estimation of multispectral cameras^12^. In this method, an image of the random noise target is acquired and split into smaller patches to account for the spatially variant nature of the PSF. For each patch, the optical transfer function (OTF) is estimated in the frequency domain using a digital ground truth template of the noise target. The PSFs are derived from the estimated OTFs. To ensure smooth spatial variation and noise-free PSF estimation, regularization of the estimated PSFs, mean and median filtering, and thresholding are performed. Compared to our method, there are some similarities. We also use a digital ground truth template, albeit not a random noise target but a text page with black letters on a white background, which makes the image registration of the digital template easier. However, we do not estimate the OTFs. Instead, we generate a noise-free PSF estimate by utilizing the computed wavefront for only a small number of spatial positions. To ensure spatially smooth variation of the PSFs, we interpolate the computed wavefront for all remaining patches where we did not perform a PSF estimation. This way, a smaller number of PSF estimations are required and no additional post-processing of the PSFs is necessary.

In the context of 3D fluorescence microscopy, Maalouf et al. proposed a method that interpolates depth-varying PSFs using only a limited number of measured PSFs^20^. They model the PSFs directly using Zernike polynomials, specifically employing 45 Zernike polynomials for the modeling. This approach differs from ours, where we use Zernike polynomials to model a wavefront that is then converted into a PSF. Our method requires significantly fewer Zernike polynomials to accurately represent PSFs formed by typical image aberrations.

To estimate the turbulence-degraded PSF from a single intensity image, Siddik et al. use a convolutional neural network to predict a modified set of Zernike polynomial coefficients which are then used to generate the corresponding PSF^21^. However, they considered only space-invariant PSFs, which usually do not apply to camera-based imaging systems.

The novelty in our proposed method lies in the use of computed wavefronts for PSF estimation on only a few spatially and spectrally separated image patches of a hyperspectral dataset and obtaining the remaining PSFs by interpolating the corresponding computed wavefronts both spatially and spectrally. This approach ensures, by design, a smooth spatio-spectral variation of the PSFs without the need for additional regularization steps.

## Methods

### Overview

The primary objective of our method is to generate a set of PSFs for a HSI system, enabling the deconvolution of spectral imaging data and thereby enhancing spatial resolution across all wavelengths. To account for the spatial and spectral variability of the PSF, we divide each frame (spectral division) of the HSI data into smaller patches (spatial division) and estimate the PSF for each individual patch (see “Description of the used HSI system and image division into patches” Section). We describe PSFs using computed wavefronts represented by Zernike polynomials, which are commonly used to model optical wavefront aberrations. These wavefronts can easily be transformed into PSFs (see “Describing optical aberrations with wavefronts and calculating the PSF” Section). The wavefront representation offers the advantage of accurately describing the PSFs of optical systems with circular apertures using only a few Zernike polynomial coefficients. By adjusting these coefficients, the shape of the wavefront-and consequently the PSF-changes. We leverage this ability to iteratively optimize the PSF estimate using an optimization algorithm. This algorithm involves performing a deconvolution with the current PSF and comparing the deconvolved patch with the corresponding ground truth patch (see “Optimization algorithm for PSF estimation” Section). The optimization algorithm is implemented in a custom software solution and applied to data from a commercial HSI system (see “Technical implementation of the optimization algorithm” Section). Furthermore, we validate the PSF estimates by comparing them to measured PSFs obtained from a custom build array of point-like light sources (see “Measuring the PSF and the spatial resolution” Section).

### Description of the used HSI system and image division into patches

In this work, we utilize the HSI system *BlackBox* by HAIP Solutions GmbH, Germany. This system operates as a spatial scanning system, also known as a push-broom system, acquiring one spatial line at a time. The imaging data has a resolution of 640 pixels by 480 pixels per frame, with a total of 501 frames per acquisition. The frames cover a wavelength range from 500 to 1000 nm, corresponding to a sampling interval of 1 nm per frame, with a spectral resolution of approximately 5 nm. To enable spatially varying PSF estimation, we divide each frame into 48 patches, each with a resolution of 80 pixels by 80 pixels. This patch size is comparable to that used by Brauers et al., who employed patches of 96 pixels by 80 pixels and achieved reasonable results^12^. However, for deconvolution, we extend each patch by adding a 10-pixel-thick border around it. After deconvolution, each patch is cropped back to its original size. These overlapping borders help avoid discontinuities at the edges of adjacent patches, particularly when large tip or tilt Zernike coefficients are present. Although these two Zernike polynomials are not responsible for any image aberrations, they shift the patch laterally and thus compensate for any mismatch between the image and the ground truth. This is crucial since our HSI system exhibits a slight wavelength-dependent spatial shift.

### Describing optical aberrations with wavefronts and calculating the PSF

Aberrations in imaging systems can be described using the concept of wavefronts, which are surfaces that represent the phase of an optical wave as it propagates through an imaging system. Wavefronts can be decomposed into a set of Zernike polynomials, which provide a mathematical description of circular wavefront profiles associated with common optical aberrations. To specify how much each Zernike polynomial $Z_{n}(r, \theta )$ contributes to the overall aberration $W(r,\theta )$ each polynomial can be multiplied with a coefficient $c_{n}$:

1 $$\begin{aligned} W(\rho ,\theta ) = \sum _{n=1}^{N}c_{n} Z_{n}(\rho , \theta ) \end{aligned}$$

Polar coordinates $\rho$ and $\theta$ are used to represent the position on the circular wavefront. *n* represents the Noll index of the current Zernike polynomial, and *N* represents the total number of Zernike polynomials used. An exhaustive overview of Zernike polynomials including detailed information about the Noll numbering scheme can be found in^22^. Zernike polynomials are orthogonal over a unit circle, making them an ideal basis for representing wavefront aberrations in circular apertures. The orthogonality allows for the separation of different types of aberrations. Each polynomial is associated with a specific type of aberration, such as defocus, astigmatism, coma, or spherical aberration. The coefficients $c_n$ in Eq. (1) quantify the contribution of each aberration type to the overall wavefront error.

The values for $c_{n}$ are generated using the method described in the subsequent section, rather than obtained from a direct wavefront measurement. Consequently, $W(\rho , \theta )$ may not accurately represent the actual wavefront errors of the optical system. Therefore, in this work, we refer to $W(\rho , \theta )$ as the *computed wavefront*. To derive the PSF from the computed wavefront, we utilize a well-established method^23^. First, the complex pupil function is constructed:

2 $$\begin{aligned} P(\rho ,\theta ) = A(\rho , \theta ) \cdot e^{-iW(\rho ,\theta )} \end{aligned}$$

The phase factor $e^{-iW(\rho ,\theta )}$ describes the phase shift introduced by the computed wavefront. $A(\rho , \theta )$ is the complex-valued amplitude transmission function, representing both the amplitude and phase modulation of the wavefront as it passes through an optical element. In our case it simply describes a circular aperture with radius $r_0$, which can be represented as:

3 $$\begin{aligned} A(\rho ,\theta ) = {\left\{ \begin{array}{ll} 1, & \text {if } \rho \le r_0 \\ 0, & \text {if } \rho > r_0 \end{array}\right. } \end{aligned}$$

Once the complex pupil function $P(\rho , \theta )$ has been obtained, the PSF of the optical system can be derived by calculating the squared modulus of the Fourier transform of the complex pupil function:

4 $$\begin{aligned} \textrm{PSF}(u, v) = \left| \mathscr {F}\{P(\rho , \theta )\}\right| ^2 \end{aligned}$$

Equation (4) represents the intensity profile of the PSF at the position (*u*, *v*) on the image. The size and shape of the PSF are determined by the wavefront aberrations induced by the optical system. Conceptually, the PSF of an optical system is the image that would be obtained when imaging a point-like object.

### Optimization algorithm for PSF estimation

We aim to optimize the coefficients of a set of Zernike polynomials that describe a digital wavefront. Our goal is to create a PSF from this wavefront, which, when used to deconvolve the input image, results in the best possible deconvolved image according to the chosen metric. In this work, we utilize the normalized cross-correlation coefficient as the image quality metric, defined by:

5 $$\begin{aligned} \text {NCC}(I_1, I_2) = \frac{\sum _{x, y} \left( (I_1(x, y) - \bar{I_1}) (I_2(x, y) - \bar{I_2}) \right) }{\sqrt{\sum _{x, y} (I_1(x, y) - \bar{I_1})^2 \sum _{x, y} (I_2(x, y) - \bar{I_2})^2}} \end{aligned}$$

where $I_1(x,y)$ and $I_2(x,y)$ represent the pixel values of the images $I_1$ (deconvolved image) and $I_2$ (ground truth image), respectively, at the coordinates (*x*, *y*), and $\bar{I_1}$ and $\bar{I_2}$ are the mean values of $I_1$ and $I_2$, respectively.

The utilized Zernike polynomial set includes the polynomials from Noll index 2 to 8, which correspond to the optical aberrations: tip, tilt, defocus, oblique primary astigmatism, vertical primary astigmatism, vertical coma, and horizontal coma.

As optimization algorithm we use Simulated Annealing^24^, which is well suited for high-dimensional optimization problems and can avoid getting trapped in local optima. The idea behind Simulated Annealing is roughly based on the cooling process of a material, where the material is heated to a high temperature and then slowly cooled down, allowing the atoms to arrange in a state with minimal energy.

The algorithm starts with an initial solution, consisting of the initial values for the Zernike coefficients $$\textbf{c} = \begin{bmatrix}c_{1}\;c_{2}\;\dots \;c_{n}\end{bmatrix}$$, and explores the search space by generating new solutions through random, minimal changes to these coefficients. To create a new solution, we use a constant perturbation factor $\epsilon$ and generate new Zernike coefficients $c'_i$ such that $c'_i = \text {rand}(c_i - \epsilon , c_i + \epsilon )$, where $\text {rand}(a, b)$ is a uniform random number in the interval [*a*, *b*].

The probability of accepting a new solution is 1 if the new solution leads to a better quality metric value than the current one; otherwise, it is given by $P_{\text {Accept}} = \exp ((Q - Q') / T)$, where *Q* and $Q'$ represent the quality metrics of the current and new solutions, respectively, and *T* is a control parameter referred to as temperature, analogous to the physical temperature in an annealing process. The temperature parameter controls the level of exploration in the search space and gradually decreases during the optimization process. This is achieved by multiplying it by a constant cooling factor $\alpha$, where $\alpha \in (0, 1)$, in each iteration step. In practice, this means that worse solutions are more likely to be accepted at the beginning of the optimization process when the temperature parameter is still high. As the optimization progresses, the temperature decreases, and at some point, only better solutions are likely to be accepted. The algorithm terminates when a predefined end temperature is reached. A summary of the optimization process in pseudocode is provided in Algorithm 1.

**Algorithm 1:**
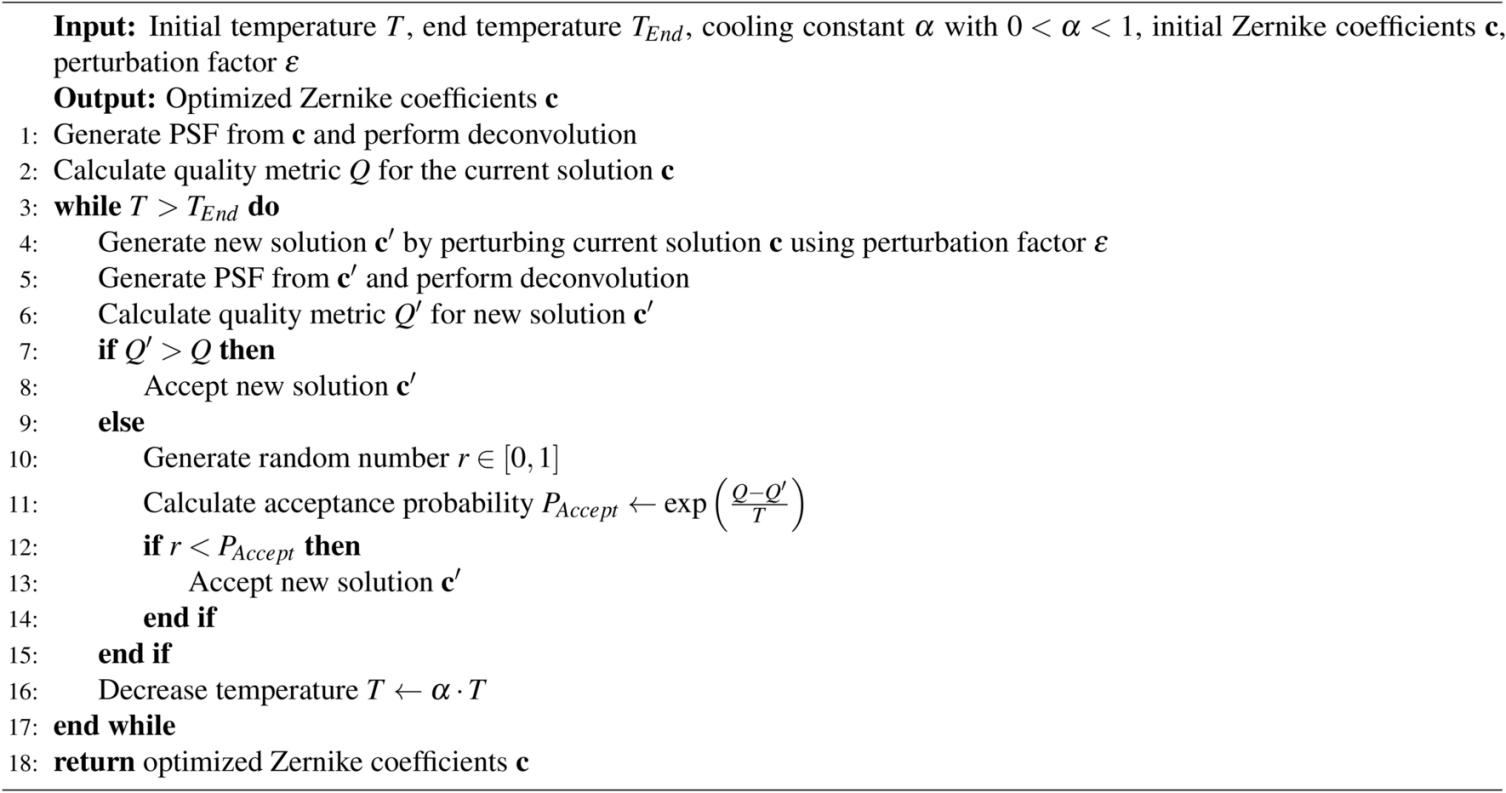
Simulated Annealing for PSF estimation with computed wavefronts. This algorithm maximizes a quality metric by iteratively perturbing the solution vector $\textbf{c}$, which contains the Zernike polynomial coefficients used to compute a wavefront for PSF generation.

This optimization is performed on every 50th frame within the range from 550 to 950 nm and only on nine key patches per frame, specifically: four corner patches, four central edge patches, and one central patch, as illustrated in Fig. 1. Consequently, out of a total of 24,048 patches, the optimization algorithm was utilized on only 91 patches. For the remaining patches, PSFs are estimated by interpolating the Zernike polynomial coefficients using third-order polynomials fitted to the corresponding Zernike coefficients in both spatial and spectral dimensions. This approach ensures a smooth transition of the PSF from one position to another and significantly reduces the overall PSF estimation time. A smooth PSF transition is essential, as abrupt changes are typically not expected in conventional optical camera systems.

We use a piece of paper with printed text as the reference image because text with Roman letters contains edges in all directions. This enables our optimization algorithm to accurately recover the PSF characteristics across all orientations. Furthermore, using a text template simplifies the generation of a ground truth image, as it is easy to visually verify alignment with the original image. In general, other types of templates with edges oriented in all directions could also be utilized.

### Technical implementation of the optimization algorithm

We have developed the PSF estimation procedure using C++ and Qt 5^25^. To compute the discrete Fourier transform for the PSF calculation from a wavefront, we utilize FFTW version 3.3.10^26^. For image metric calculation and deconvolution, we employ the parallel computing library ArrayFire v3.8.2^27^, which provides built-in deconvolution algorithms such as Richardson–Lucy, Tikhonov, and Landweber. Additionally, we have implemented Wiener deconvolution. While different deconvolution algorithms showed no significant difference in the resulting PSFs, we chose the Richardson–Lucy method for its control over the number of iterations, allowing a trade-off between sharpness, noise and computation time. In this work, we used 128 iterations. Polynomial fitting for wavefront interpolation is achieved using Eigen 3.4.0^28^.

For easy interaction and visualization of the results we implemented a graphical user interface. In addition to optimization using Simulated Annealing, the software also allows for manual adjustment of the Zernike coefficients, i.e. the digital wavefront, to generate PSFs and apply them in deconvolution. The deconvolved image is displayed without noticeable delay, enabling intuitive exploration of the solution space. This is particularly useful for generating an initial solution for the Simulated Annealing optimization. For the central key patch of the first frame, we manually adjust the coefficients for defocus and astigmatism until a noticeable improvement in image quality is observed. This manually adjusted wavefront is then used as the initial solution for the Simulated Annealing optimization of the same patch. The resulting optimized wavefront is subsequently used as the starting wavefront for the remaining patches within the same frame. For patches in subsequent frames, we use wavefronts from the patches of adjacent frames as their initial wavefronts.

A video illustrating the optimization process of a single patch can be found in the supplementary materials (see Supplementary Movie 1). In the video, the image metric plot displays the NCC multiplied by − 100 instead of the NCC itself. Converting this maximization problem to a minimization problem was advantageous during software development, as it allowed for testing other image metrics that require minimization to achieve optimal results. Please note that the speed of the video has been increased; in reality, an optimization process involving 748 iterations, as shown in the video, takes approximately 100 seconds on the hardware used in this study (Intel®Core^TM^ i7-10750H CPU @ 2.60GHz, NVIDIA Quadro P620).

### Measuring the PSF and the spatial resolution

**Fig. 2:**
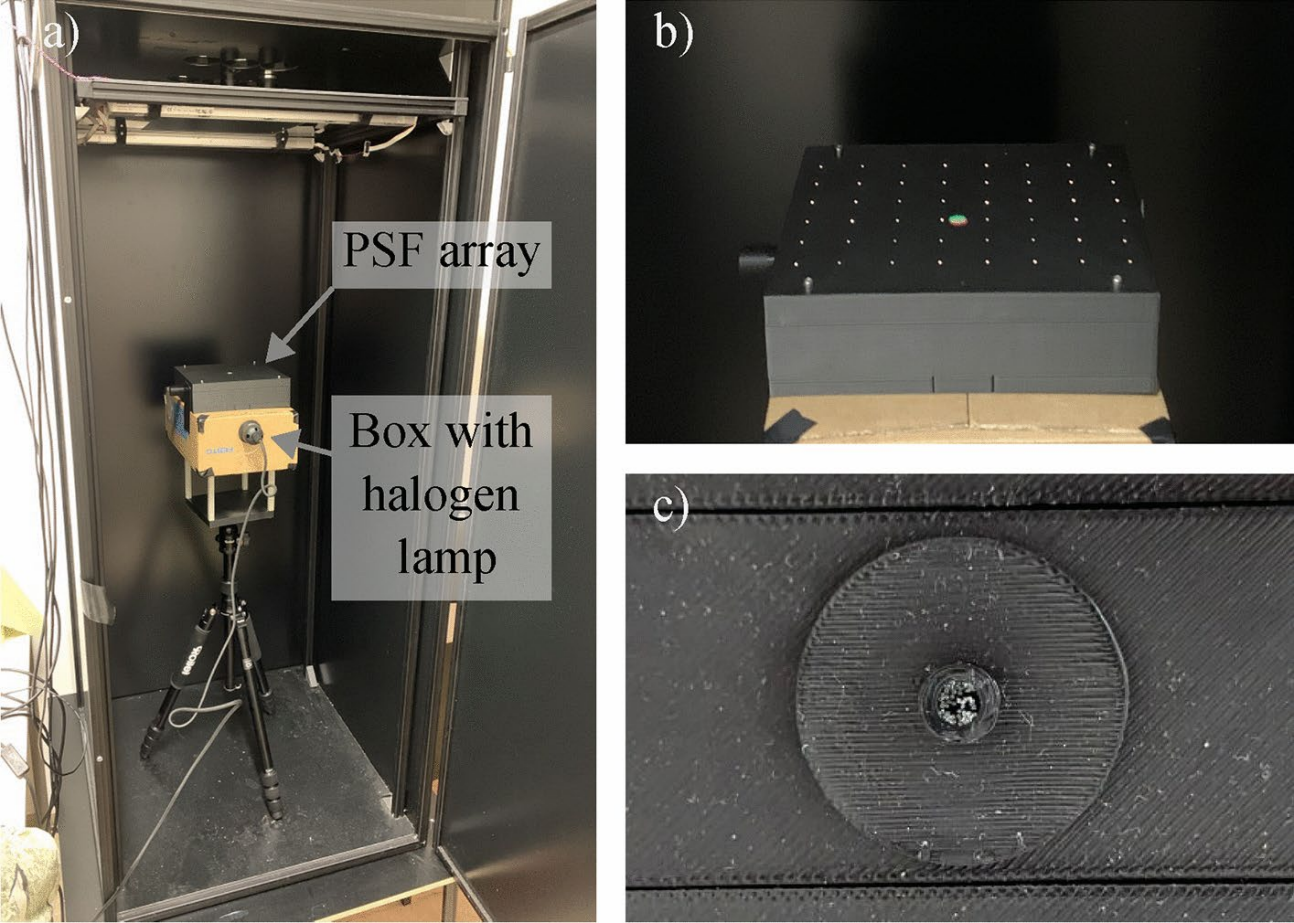
The PMMA light guide-based array of point-like light sources used for PSF measurement. (**a**) The PSF measurement setup inside the HSI system (*BlackBox* by HAIP Solutions GmbH, Germany). The halogen lamp (not visible in the pictures) is inside a box to block light from the sides. (**b**) View of the point-like light sources with the halogen lamp enabled. The green and red spots in the center of the PSF measurement unit are distance alignment light spots from the HSI system. (**c**) Fiber bundle at the bottom of the PSF array box. When in use, this fiber bundle points inside the box with the halogen lamp.

For PSF measurement, we built a Polymethyl methacrylate (PMMA) light guide-based array of point-like light sources, see Fig. 2. We chose fibers with a diameter of 250 $\upmu$m. This is smaller than the size represented by one pixel, which is approximately 300 $\upmu$m, and smaller than the best optical resolution, across the entire spectral range, in the center of view, which is approximately 360 $\upmu$m. The array consists of 48 light guides, bundled on one side to create a single input for a halogen light source. The output is arranged evenly in an 6 by 8 pattern, matching the pattern of the PSF optimization regions. The fibers are large enough to provide sufficient light for HSI acquisition, However, for PSF acquisition, the integration time needed to be increased from 6,6 to 66  ms, which required a reduction in the spatial scanning speed for image acquisition. Before using the measured PSFs for deconvolution, contrast adjustment and normalization are performed for each PSF separately to ensure easy comparability between different PSFs.

To analyze the spatial resolution of the system, an image of the 1951 USAF resolution test chart was acquired and evaluated using the ASI_MTF plugin^29^ for ImageJ. This plugin calculates the Modulation Transfer Function (MTF) as a function of pixel spatial frequency. The resolution is defined as the distance corresponding to the spatial frequency at which the MTF curve drops to a contrast value of 26.4 % (Rayleigh criterion)^30^. Using this criterion and the known sizes of the elements in the USAF test chart, we determined the spatial resolution in millimeters for each frame of the HSI data set in both horizontal and vertical directions.

## Results

### Comparison between estimated and measured PSFs

**Fig. 3:**
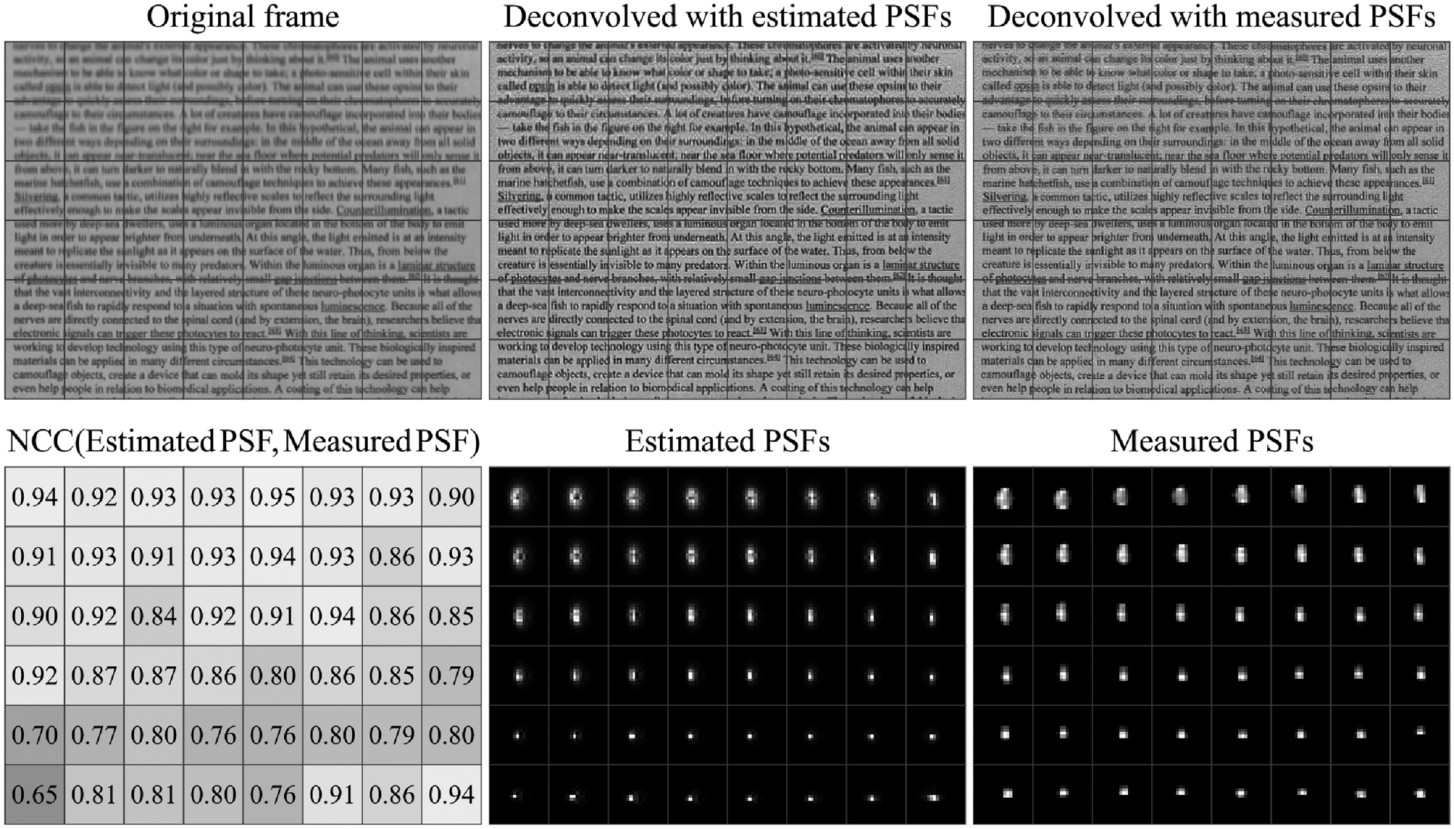
Comparison of estimated and measured spatially varying PSFs. The top row shows single frames at 700 nm divided into 6 by 8 patches. The first frame shows the original unprocessed reflectance frame. The next two frames are deconvolved with estimated PSFs and measured PSFs, respectively. In the first frame of the bottom row, the normalized cross-correlation coefficient (NCC) is used to quantify the similarity between the estimated and measured PSFs. An NCC value of 1.0 indicates that the PSFs are identical, whereas a value of 0.0 indicates no correlation. The subsequent frames display the estimated PSFs and measured PSFs for each patch of the frame, respectively. The HSI images show a portion of a printed page with text sourced from a Wikipedia article^31^.

We evaluate the accuracy of the estimated PSFs by quantifying the similarity between the estimated and actual PSFs of the HSI system. This evaluation employs the NCC, where values approaching 1.0 indicate higher similarity, and a value of 0.0 signifies no correlation. The actual PSFs were measured using a custom-built, fiber-based array of point-like light sources. To account for the spatial variance of the PSF, each frame is divided into a 6 by 8 grid of patches for PSF estimation, measurement, and deconvolution. Figure 3 presents the estimated and measured PSFs for a single frame at 700 nm.

**Fig. 4:**
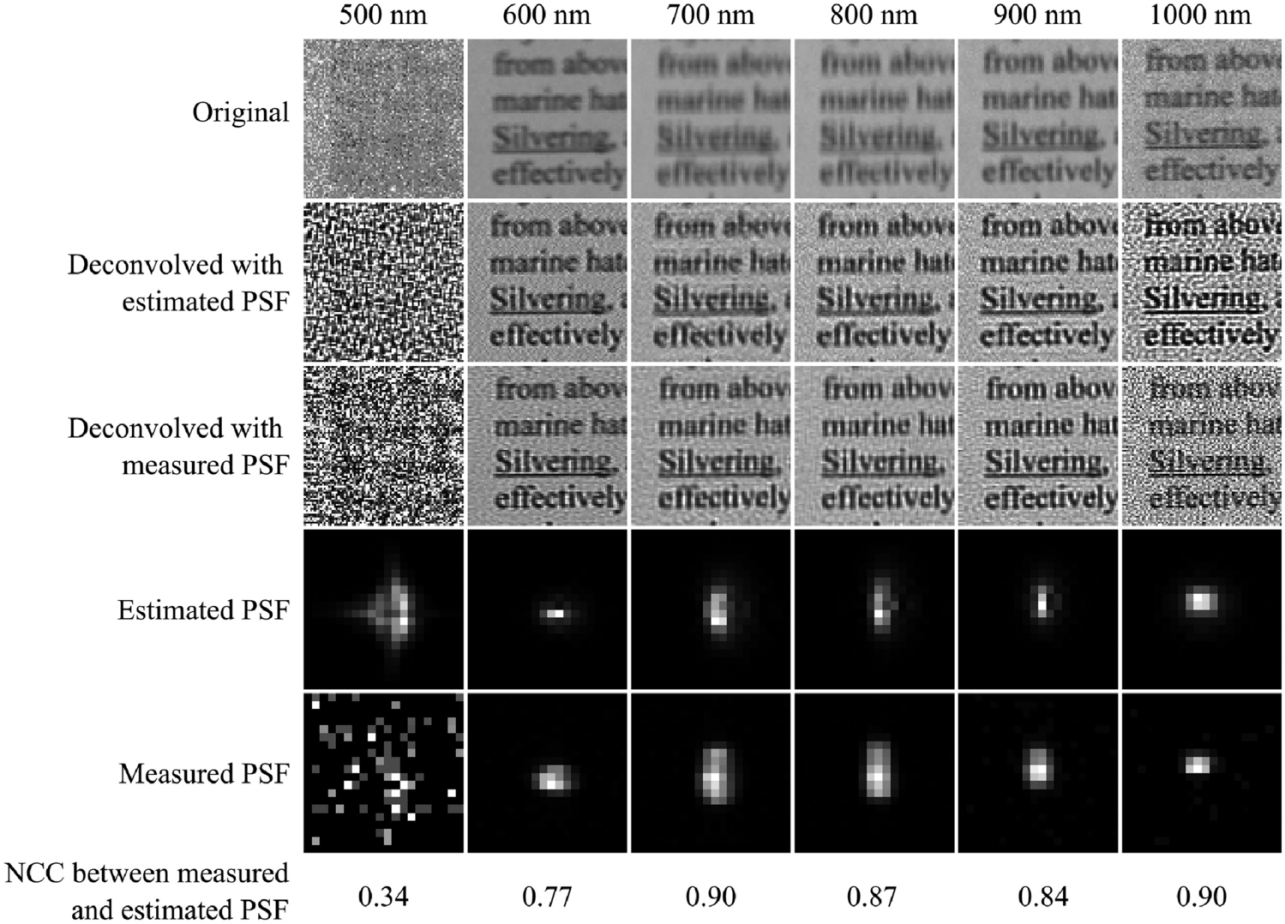
Comparison of estimated and measured spectrally varying PSFs. At the beginning of the spectral measuring range of the HSI system, the noise is too high for any meaningful PSF estimations or measurements. The deconvolution with the estimated and measured PSFs results in similar-looking images.

**Fig. 5:**
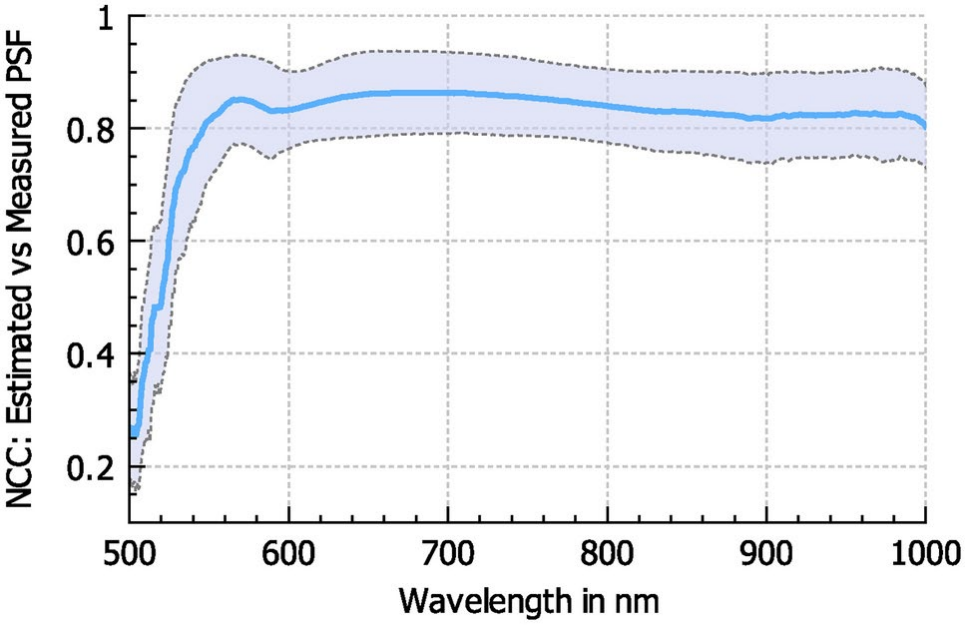
Normalized cross-correlation coefficient between estimated and measured PSFs of all patches per frame for the full spectral range of our HSI system. The bold blue line shows the mean value of the NCC, and the dotted lines represent a distance of one standard deviation from the mean value. Due to the high noise level for the wavelengths from 500 to 525 nm, no PSF could be measured for this region, which correlates to very low NCC values at the beginning of the plot. Starting at 550 nm, the noise level in the measured PSFs is much lower, and the measured and estimated PSFs show a high degree of similarity, with NCC mean values above 0.8.

Figure 4 compares the PSFs of a single patch across different wavelengths. The NCC is also used here to quantify the similarity between the estimated and measured PSFs. The first 25 frames of the HSI data are too noisy for any meaningful PSF estimation or measurement, resulting in very low NCC values for the wavelength range of 500–525 nm in Fig. 5. For all other wavelengths, the PSFs show a high similarity to the measured PSFs.

### Spatial resolution

**Fig. 6:**
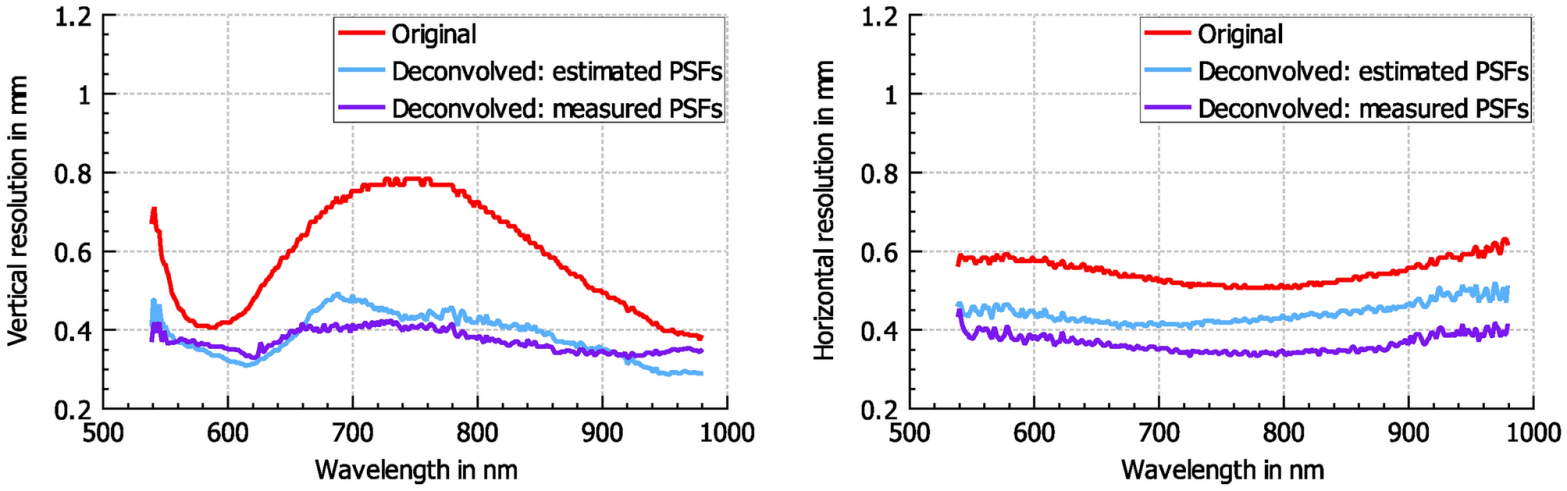
Spatial resolution at the center position of the frame for each wavelength. Left: resolution in vertical direction. Right: resolution in horizontal direction.

**Fig. 7:**
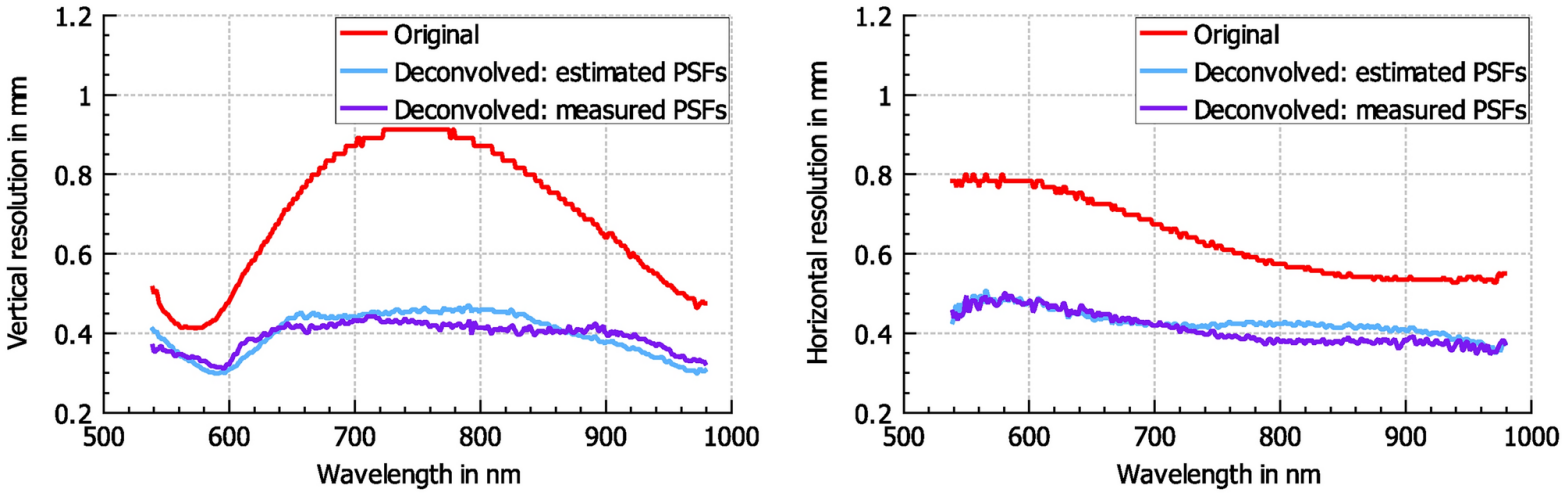
Spatial resolution at the middle upper edge area of the frame for each wavelength. Left: resolution in vertical direction. Right: resolution in horizontal direction.

Figure 6 shows the spatial resolution, based on the Rayleigh criterion, in both vertical and horizontal directions for the center region of the HSI field of view for each wavelength. The comparison includes the unmodified original data set, the data set deconvolved with estimated PSFs, and the data set deconvolved with measured PSFs. The spatial resolution improves for all wavelength after deconvolution. Comparable results were achieved at the edges of the HSI field, whereby the original resolution was in some cases worse than 900  $\upmu$m and could be improved to approx. 450  $\upmu$m by deconvolution. The wavelength-dependent spatial resolution for the at the middle upper edge area of the HSI field of view can be seen in Fig. 7. The mean resolution across all wavelengths for the original and deconvolved data can be seen in Table 1.

**Table 1:** Mean values and standard deviations of spatial resolution in vertical and horizontal directions for different regions of the field of view across all wavelengths. Results are shown for the original data, data deconvolved with estimated PSFs, and data deconvolved with measured PSFs.

Region	Direction	Resolution of original image data (mm)	Resolution after deconvolution with estimated PSFs (mm)	Resolution after deconvolution with measured PSFs (mm)
Center area	Vertical	0.59 ± 0.14	0.39 ± 0.06	0.37 ± 0.03
	Horizontal	0.55 ± 0.04	0.44 ± 0.03	0.37 ± 0.03
Middle upper edge area	Vertical	0.70 ± 0.17	0.40 ± 0.06	0.39 ± 0.04
	Horizontal	0.63 ± 0.10	0.43 ± 0.04	0.41 ± 0.05

### Number of Zernike polynomials for PSF estimation

**Fig. 8:**
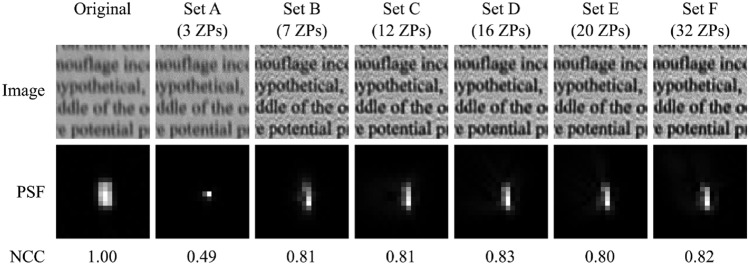
Results of the PSF estimation process for different numbers of Zernike polynomials (ZPs), see Table 2 for a list of the used polynomials. The first column shows the original image and the corresponding measured PSF, which was compared to the estimated PSFs via NCC calculation. These estimated PSFs were also used to deconvolve the original image. The “Image” row shows for each estimated PSF the corresponding deconvolved image.

To investigate how many Zernike polynomials are required to appropriately describe the PSFs in our HSI system, we performed the PSF estimation with different sets of Zernike polynomials, see Table 2. Only seven polynomials (from Noll index 2 to 8) are sufficient to achieve a PSF estimation that is close to the measured PSF. Sets with more Zernike polynomials did not significantly improve the result, as can be seen in Fig. 8.

**Table 2:** Zernike polynomial sets used to compare resulting PSFs with measured PSFs, see Fig. 8.

Set name	Zernike polynomials (noll indices)	Corresponding aberrations
Set A	2 to 4	Tip, Tilt, Defocus
Set B	2 to 8	Set A + Oblique Primary Astigmatism, Vertical Primary Astigmatism, Vertical Coma, Horizontal Coma
Set C	2 to 13	Set B + Vertical Trefoil, Oblique Trefoil, Primary Spherical, Vertical Secondary Astigmatism, Oblique Secondary Astigmatism
Set D	2 to 17	Set C + Vertical Quadrafoil, Oblique Quadrafoil, Horizontal Secondary Coma, Vertical Secondary Coma
Set E	2 to 21	Set D + Oblique Secondary Trefoil, Vertical Secondary Trefoil, Oblique Pentafoil, Vertical Pentafoil
Set F	2 to 33	Set E + the next 12 higher-order aberrations

### Comparison between original and deconvolved images and reflectance spectra

**Fig. 9:**
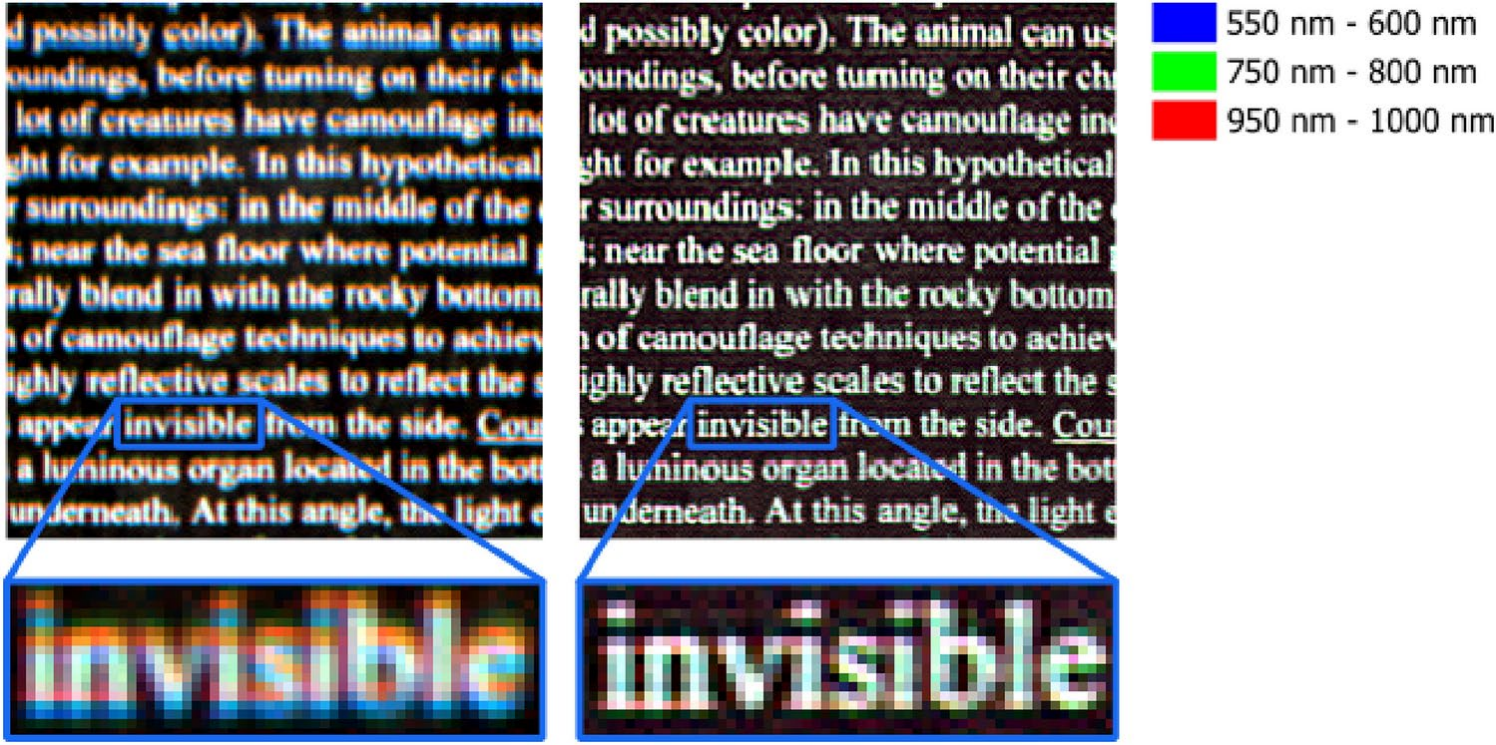
Comparison of wavelength-dependent spatial shift between the original dataset (left) and the deconvolved dataset using the estimated PSFs (right). Three spectral regions, each with a 50 nm bandwidth, are averaged, color-coded, and overlaid. Using the estimated PSFs for deconvolution spatially aligns all frames, minimizing the spatial shift.

Acquisitions from our HSI system exhibit a wavelength-dependent spatial shift, which can compromise the accuracy of spatio-spectral information extraction and processing. Our proposed method corrects this shift, as visualized in Fig. 9. The deconvolved dataset shows significantly improved spatial alignment across different wavelengths.

**Fig. 10:**
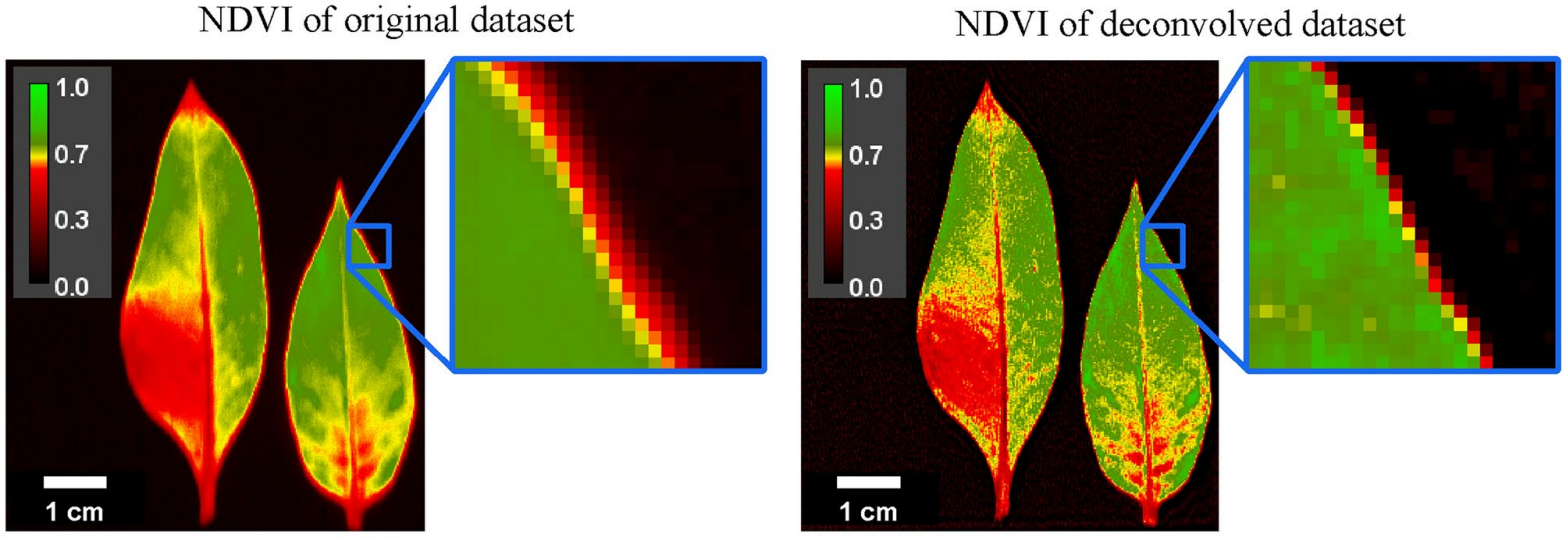
Comparison of NDVI for the original dataset (left) and the deconvolved dataset (right). The NDVI based on the original dataset exhibits an artificial halo around the edges of the tobacco leaves due to the difference in optical resolution of the red and near-infrared reflectance data. This image artifact is substantially reduced when using the deconvolved dataset for NDVI calculation.

A common method that utilizes spatio-spectral properties of HSI data to extract specific information is the Normalized Difference Vegetation Index (NDVI)^32,33^. This is a widely used metric that quantifies plant health and vigor by using information from the visible red range (e.g., at 680 nm) and from the near-infrared range (e.g., at 750 nm). It is calculated by dividing the difference between the red and near-infrared reflectance values by the sum of these reflectance values. However, since the spatial resolution in the near-infrared is usually slightly worse compared to the resolution in the red spectral range, the spatial information in the reflectance images does not match perfectly. This introduces a halo-like image artifact around edges which can be seen in the left part of Fig. 10. After deconvolution of the hyperspectral dataset, the resulting NDVI image shows a greatly reduced halo artifact, and finer details of the leaves are discernible (Fig. 10 right).

**Fig. 11:**
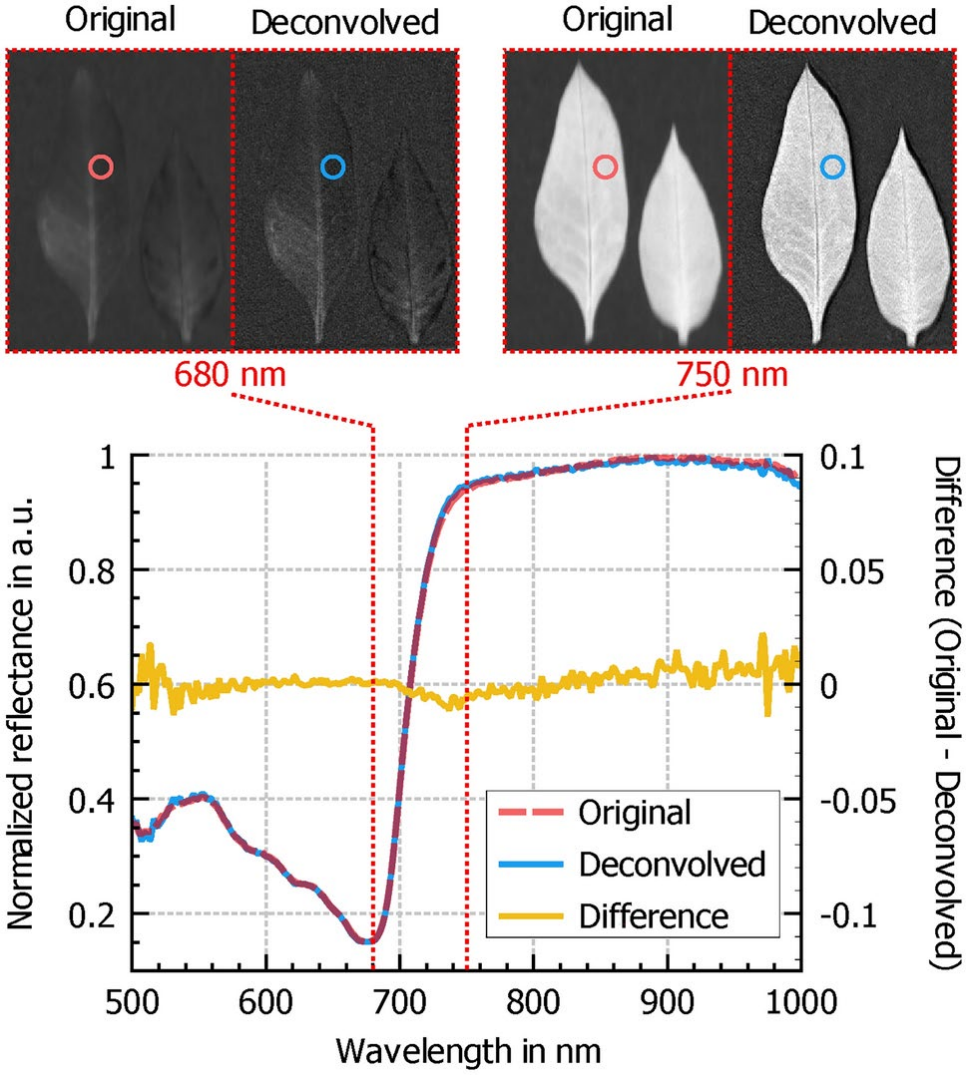
Comparison of spectra for original and deconvolved data. Top left: Original and deconvolved frames at 680 nm, with circles indicating the areas used to extract the average spectra of all enclosed pixels. Top right: Original and deconvolved images at 750 nm. Bottom: Plot of the average spectra (left axis) for the original and deconvolved data. The difference between the two spectra (right axis) is close to zero across the entire wavelength range.

It is essential that the deconvolution process preserves spectral information when using the deconvolved data for calculating vegetation indices and performing other spectral analyses. Figure 11 presents the spectra of the original and deconvolved data sets from one of the tobacco leaves and their difference. The maximum difference between both spectral lines is less than 2.2% of the total value range.

## Discussion

In this work, we demonstrated that computed wavefronts, based on Zernike polynomials combined with an optimization algorithm, can be used for accurate PSF estimation. Utilizing the estimated PSF for deconvolution significantly improved the spatial resolution of the hyperspectral data. To address the spatial and spectral variations of the PSF, we divided the hyperspectral data into patches, with each patch having a distinct PSF. We found that a small number of Zernike polynomials are sufficient to accurately describe a PSF in the HSI system we used. Moreover, only 91 out of 24,048 patches required PSF estimation via an optimization algorithm. The remaining PSFs are generated by interpolating the computed wavefronts. This interpolation approach reduces computation time and ensures a smooth transition of the PSFs. One significant advantage of our method is that the generated PSFs are free of noise. In contrast, other approaches that measure the response of specialized targets for PSF estimation^12,13^ require an additional noise reduction step before the estimated PSFs can be used for deconvolution. Using our estimated PSFs to deconvolve subsequent HSI images greatly improved spatial resolution while keeping the spectral information intact.

The resulting spatial resolution after deconvolution, using the estimated PSFs, is significantly improved. The original resolution varies both spatially and spectrally, and in some cases, it is worse than 900 $\upmu$m. After deconvolution, a more homogeneous spatial resolution is achieved, approximately 450 $\upmu$m, in both spatial and spectral directions. The resolution achieved using the estimated PSFs matches that attained with the measured PSFs in the vertical direction. In the horizontal direction, the measured PSFs yield a slightly better resolution. However, it is important to note that with the measured PSFs, deconvolution could not be successfully performed for all patches. This limitation arose because, at certain spatial and spectral locations, the noise levels were too high to recover a PSF, making deconvolution impossible at these positions.

A limitation of our approach is that the two-dimensional PSFs can only be used for deconvolution to improve spatial resolution. These spatial PSFs do not capture information about optical aberrations in the spectral direction. To address this, an additional spectral PSF measurement or estimation could be performed, for example, by using a monochromator as a light source, as demonstrated by Jemec et al.^7^.

Another limitation is the inherent trade-off between improved spatial resolution and amplified noise following deconvolution. While deconvolution enhances resolution, it can also increase noise and introduce ringing artifacts around edges. The number of iterations in the Richardson–Lucy algorithm provides a means to balance resolution improvement and noise amplification. However, the acceptable noise levels are highly dependent on the specific application and may require a quantitative analysis to determine appropriate parameters for each case.

Despite the promising results, our approach has some room for improvement that needs to be addressed in future studies. One crucial aspect is that the computed wavefronts, constructed using Zernike polynomials, do not necessarily represent the actual optical aberrations of the imaging system. Identical PSFs can be generated from different wavefronts^34^. In the worst-case scenario, two widely differing computed wavefronts in close spatial or spectral proximity can result in completely unsuitable interpolated wavefronts between them. A simple example for this issue is the sign of the defocus polynomial, where identical PSFs can be produced with both negative and positive defocus coefficients, with the remaining polynomial coefficients having opposite signs. To mitigate this, we constrain the defocus polynomial to only accept negative coefficients. However, when an optical system requires a high number of Zernike polynomials for accurate PSF estimation, the potential for these ambiguities increases. A straightforward approach to reduce the generation of widely differing wavefronts due to wavefront-PSF ambiguity is to use the wavefront of a previously estimated PSF as the initial solution for other patches in the frame. Additionally, employing optimization algorithms that restrict the solution space and adopt a greedy strategy-always selecting the immediate better solution-can help. However, this increases the risk of getting stuck in local maxima, resulting in suboptimal PSF estimates. There are several potential avenues for future research that could enhance the performance and applicability of our method. For example, alternative approaches to compute wavefronts beyond Zernike polynomials exist, such as simulating the shape of a deformable mirror with actuators beneath a continuous membrane surface. This could improve the resulting PSFs in systems where the assumption of circular wavefront profiles is inadequate. Additionally, it would be highly desirable to use an image quality metric that does not require a ground truth image as a reference. This advancement would transform our proposed method into a truly blind deconvolution technique. Another aspect that warrants further investigation is the determination of optimal patch size. In our study, the patch size was selected based on the sizes used in similar approaches found in the literature^12–14^. Conducting a systematic investigation into the optimal patch size could potentially lead to better deconvolution results.

In conclusion, we have introduced a novel method for PSF estimation in HSI systems. The use of estimated PSFs for patchwise deconvolution has demonstrated improvements in spatial resolution across all wavelengths and a reduction of the wavelength-dependent spatial shift in the image stack.

## Supplementary Information

Supplementary Information.

## Data Availability

The datasets generated and analyzed during this study are publicly available in the Research Data Repository of Leibniz University of Hannover at https://doi.org/10.25835/yu47lho4.
